# Clinical Features and Factors Associated with Outcomes of Patients Infected with a Novel Influenza A (H7N9) Virus: A Preliminary Study

**DOI:** 10.1371/journal.pone.0073362

**Published:** 2013-09-17

**Authors:** Xiaorong Chen, Zongguo Yang, Yunfei Lu, Qingnian Xu, Qiang Wang, Liang Chen

**Affiliations:** 1 Department of Traditional Chinese Medicine, Shanghai Public Health Clinical Center Affiliated to Fudan University, Shanghai, China; 2 Key Laboratory of Infectious Diseases of State Administration of Traditional Chinese Medicine (Clinical base), Shanghai, China; 3 Department of Hepatitis, Shanghai Public Health Clinical Center Affiliated to Fudan University, Shanghai, China; D'or Institute of Research and Education, Brazil

## Abstract

**Objective:**

The present study aimed to analyze clinical features and factors associated with treatment outcomes of H7N9 influenza A virus infection.

**Methods:**

The clinical progress in 18 H7N9-infected patients was monitored and recorded. The clinical features of H7N9 infection were noted and factors associated with treatment outcomes were analyzed by univariate analyses.

**Results:**

The average ages of patients in recovered and critical conditions were 67.0±10.83 years and 72.75±12.0 years, respectively. Renal insufficiency developed more frequently in critically ill patients (*P* = 0.023). The duration of traditional Chinese medicine (TCM) therapy was longer in recovered patients than in critically ill patients (*P* = 0.01). Laboratory tests showed that levels of C-reactive protein, serum creatinine, and myoglobin were significantly higher in critically ill patients than in recovered patients (*P* = 0.011, 0.04, and 0.016, respectively). Meanwhile, levels of all T cell subsets examined including total CD3^+^, CD4^+^, CD8^+^, and CD45^+^ T cells were lower in critically ill patients than in recovered patients (*P* = 0.033, 0.059, 0.015, and 0.039, respectively). Logistic regression analysis demonstrated that C-reactive protein level, myoglobin level and TCM therapy duration were likely associated with treatment outcomes of H7N9 infection (*P* = 0.032, 0.041 and 0.017, respectively).

**Conclusion:**

Elderly people may have increased risk for H7N9 virus infection. T cell-mediated responses play an important role in defense against the H7N9 virus. C-reactive protein level, myoglobin level and TCM duration may be associated with treatment outcomes of H7N9 infection.

## Introduction

Avian influenza caused by influenza A viruses (IAV) has become a serious threat to human health in recent years. Infections with avian influenza viruses, which usually occur after exposure to poultry and/or wild birds, are commonly characterized by conjunctivitis, upper and/or lower respiratory tract disease, pneumonia, and multiple organ failure [1]–[3]. Wild birds are believed to constitute the natural reservoir for IAVs and play a central role in the spread of the viruses.

IAVs are classified into subtypes based on different combinations of 16 hemagglutinin (HA: H1 – H16) and 9 neuraminidase (NA: N1 – N9) surface antigens, and two pathotypes (low and high pathogenicity) based on lethality for chicken. All highly pathogenic avian IAVs known to date belong to the H5 or H7 subtype [4]. The H7N9 viruses, a subgroup of H7 viruses that normally spread among birds have currently been found to cause human infections in China, prompting intensive research to address the challenge of a potential epidemic/pandemic. Although H7N9 family clusters have been reported in Shanghai and Shandong Province [5], no evidence of human-to-human H7N9 virus transmission has been found to date [6]. Presently, there is a general lack of information about H7N9 infections, e.g., the transmission mechanism and the number of mild infections in China are unknown. Unlike H5N1, which could wipe out a flock of poultry within days, H7N9 infections in poultry are usually asymptomatic or mild. Nonetheless, based on what has been learned from the H5N1 avian flu virus which has killed 371 people in 15 countries since 2003 and proved extremely difficult to control, the risk of an H7N9 epidemic/pandemic should not be underestimated, especially in China where poultries are reared with diverse methods without strict biosecurity measures [7]. Studies on H7N9 transmission mechanisms and clinical features/outcomes are critical to prevent and control potential H7N9 epidemic/pandemic.

Overall, few cases of H7 virus transmission to mammals have ever been reported in Asia and N9 virus infections in human have never been documented anywhere in the world [8] except H7N9 [7]. The H7N9 variants currently in circulation most likely evolved through a combination of genes from viruses in Beijing bramblings, Zhejiang ducks, and Korean wild birds according to report by Chinese scientists. Human infections with highly pathogenic H7 viruses generally resulted in conjunctivitis or uncomplicated influenza [3], [8]. By April 30, 2013, confirmed cases of H7N9 infections had been reported in 11 provinces across the Yangtze Delta region of Eastern, Northern, and Southeast China. A total of 126 patients have been diagnosed with H7N9 infection and 24 patients have died since the first confirmed case was reported on April 24, 2013 in Taiwan. Among the 33 patients diagnosed in Shanghai, 21 have recovered while 12 died from complications caused by the infection, as confirmed by the Shanghai Municipal Health and Family Planning Commission. The clinical features, treatment, outcomes, and other factors associated with H7N9 infection have not been reported. From April 6 to April 20, 2013, 18 H7N9-infected patients were admitted into our hospital, which is one of the designated treatment centers for H7N9 infection in Shanghai. In the present article, we reported our observations of clinical features and clinical progress in H7N9-infected patients and interpreted factors related to treatment outcomes. The information provided herein could help early screening, diagnosis, and treatment of H7N9 infection and facilitate development of strategies to prevent death.

## Materials and Methods

### Ethics statement

Study protocol for this preliminary investigation and informed consent documents were reviewed and approved by the Ethics Committee of Shanghai Public Health Clinical Center Affiliated with Fudan University. All participants provided written informed consent after receiving information of the aims, methods, anticipated benefits, and potential hazards of the study. All work reported herein was conducted in China.

### Patients

Diagnosis of H7N9 infection in patients was made by positive test for H7N9 viral RNA. Throat-swab specimens were collected from patients and tested for H7N9 viral RNA by real-time reverse transcription polymerase chain reaction (RT-PCR), which was carried out using standard RT-PCR protocol at Shanghai Centers for Disease Control and Prevention. All 18 Patients who participated in the study were admitted to Shanghai Public Health Clinical Center and diagnosed with H7N9 infection from April 6 to April 20, 2013. Their medical records were obtained and analyzed.

Laboratory tests of blood, liver function, renal function, myocardial enzyme, T cells, and immunoglobulins were carried out at Medical Inspection Department of Shanghai Public Health Clinical Center. Other information about patients was either retrieved from patients' medical records or acquired directly from patients via a questionnaire. Factors analyzed for possible correlation with clinical features and treatment outcomes in patients included 1) baseline characteristics of patients, such as age, sex, occupation, underlying conditions, exposure to poultry and/or wild birds in the past seven days, date of symptom onset and hospital admission, date of specimen collection, and date of positive diagnosis; 2) results from laboratory tests and imaging examinations; 3) treatment regimen including basic supporting therapy, antibiotic therapy, antiviral therapy, traditional Chinese medicine (TCM) therapy, and other therapies if applicable; and 4) current condition of patients including the length of stay in the hospital.

Following guidelines from the influenza A H7N9 clinic program published by the National Health and Family Planning Commission of P. R. China (the 2nd edition, 2013) [9], we considered patients who met the following criteria recovered: 1) throat-swab specimens tested negative for H7N9 viral RNA, and 2) disappearance of fever and other clinical symptoms and signs. Following the same set of guidelines, we considered patients who met the following criteria critically ill: 1) pneumonia with two or more complications, such as acute respiratory distress syndrome, heart failure, renal failure, septic shock, encephalopathy, and secondary infections; and 2) no improvement in at least one of the above mentioned complications after 3 d of active treatment.

### Treatment

All participating patients were treated by twice daily oral administration of 75 mg oseltamivir. Patients were also given antibiotics based on blood and/or throat-swab specimens/sputum tests for bacterial infections. If no specific bacterial pathogens were detected from the specimens, advanced treatment was considered. Antibiotics given to H7N9-infected patients if applicable included moxifloxacin, sulbactam and cefoperazone, levofloxacin, meropenem, piperacillin, imipenem, and cilastatin. Some patients also received glucocorticoid therapy, intravenous immunoglobulin therapy, and TCM therapy. Only Chinese herbs prescribed according to specific syndromes were considered TCM in this study. Proprietary Chinese medicines and injections of Chinese medicines were excluded because they could contain certain western drug ingredients. These herbal TCMs were prescribed following group discussions of TCM experts from Longhua Hospital, Shanghai University of TCM, Department of TCM of Zhongshan Hospital, Fudan University, and XR Chen in our hospital. Based on syndrome differentiation criteria from *Wei-Qi-Ying-Xue* and clinic programs of influenza A H7N9 implemented by the National Health and Family Planning Commission of P. R. China, *Yinqiao Powder* and *Hoisting Powder* were prescribed for patients with mild syndromes and *Qingwen-Baidu-Decoction* was prescribed for critically ill patients. TCMs were taken orally twice daily at 150 ml decoction per dose. The purchase, decoction, and administration of Chinese herbs were supervised by Pharmacy Department of TCM in Shanghai Public Health Clinical Center.

### Statistical analysis

Baseline data were expressed as mean ± standard deviation (*SD*) or median values. The Kolmogorov Smirnov test was used for the test of normality on quantitative data. To analyze differences between baseline data, Student's *t*-test analysis was performed on mean numeric data, Mann–Whitney *U*-test analysis was performed on non-numeric data, and Fisher's exact test was used for categorical variables. Logistic regression models were used to evaluate possible factors contributing to different outcomes in H7N9-infected patients. Results were reported as odds ratios (*OR*) with their 95% confidence intervals (*CI*). All baseline characteristics and laboratory tests were analyzed using univariate analysis. Multivariate analysis was not considered suitable for such a small sample study. Statistical analyses were performed using PASW Statistics software version 18.0 from SPSS Inc. (Chicago, IL, USA). All *P* values were two-tailed, and differences with *P*<0.05 were considered statistically significant.

## Results

### Patient characteristics

Patients were grouped according to their conditions. Patients in group A were in recovered condition or had recovered, and patients in group B were in critical condition or had died. There were 14 males and four females in the 18 H7N9-infected patients. As shown in Table 1, 10 patients were in group A and eight were in group B. Average ages of patients in group A and group B were 67.0±10.83 and 72.75±12.0 years, respectively. Four patients, including three from group A and one from group B, had a history of smoking. In group A, seven patients had underlying conditions: five had hypertension, one had coronary heart disease, and one had chronic obstructive pulmonary disease (COPD). In group B, nine patients had underlying conditions: three had hypertension, two had diabetes, two had coronary heart disease, and two had COPD. Only three patients, one in group A and two in group B, had confirmed exposure to poultry and/or wild birds 7 d prior to onset of illness. Average time from onset of symptoms to diagnosis was 7.1±2.03 days in group A and 6.38±2.07 days in group B. Average time from onset of symptoms to treatment with antiviral agents was 5 or 6 days in both groups. Five patients, including one from group A and four from group B, developed acute respiratory distress syndrome during the course of the disease. Seven patients, two from group A and five from group B had heart failure. Four patients experienced renal failure and three experienced septic shock, all from group B. Three patients in group B were diagnosed with encephalitis based on their clinical symptoms, dysphoria and one in lethargy condition. All three patients developed poor cognitive ability as observed in medical examinations although no CSF test was conducted on these patients. Two patients died while the third in critical condition with no improvement observed in their neurological condition throughout treatment. Moreover, three patients from group B who tested positive in the sputum culture fungal spore test also had secondary infections. Renal failure rates were significantly different between the two groups (*P* = 0.023).

**Table 1:** Baseline characteristics of 18 H7N9-infected patients.

	Group A	Group B	
Baseline characteristics	N = 10	N = 8	*P*
Gender, N (%)			
Male	8 (80)	6 (75)	1.0
Age, years (mean ± *SD*)	67.0±10.83	72.75±12.0	0.302
Smoking history, N (%)	3 (30)	1 (12.5)	0.588
Underlying conditions, N (%)			
Hypertension	5 (30)	3 (37.5)	0.664
Diabetes	0	2 (25)	0.183
Coronary heart diseases	1 (10)	2 (25)	0.559
COPD	1 (10)	2 (25)	0.559
Exposure to poultry and/or wild birds in 7 days before illness onset	1 (10)	2 (25)	0.559
Time from illness onset to confirmed, day (mean±*SD*)	7.1±2.03	6.38±2.07	0.465
Time from illness onset to antiviral, day (mean±*SD*)	5 (4–10)	6 (3–10)	0.928
Complications, N (%)			
Acute respiratory distress syndrome	1 (10)	4 (50)	0.118
Heart failure	2	5	0.145
Renal failure	0	4	**0.023**
Septic shock	0	3	0.069
Encephalopathy	0	3	0.183
Secondary infetions	0	3	0.069
Treatment regimens, N (%)			
Oxygen therapy			
Nasal catheter	10 (100)	1 (12.5)	**<0.001**
Mask	4 (40)	4 (50)	1.0
Non-invasive ventilation	2 (20)	6 (62.5)	0.054
Tracheal intubation	0	3 (37.5)	0.069
Extracorporeal membrane oxygenation	0	2 (25)	0.183
Antiviral agent	10 (100)	8 (100)	1.0
Antibiotic therapy	10 (100)	8 (100)	1.0
Glucocorticoid therapy	6 (60)	5 (62.5)	1.0
Intravenous immune globulin therapy	6 (60)	3 (37.5)	0.637
Traditional Chinese Medicine therapy	10 (100)	4 (50)	**0.023**
Time from illness onset to TCM therapy, day (mean ± *SD*)	8.5 (5–14)	3.5 (0–10)	0.066
Duration of TCM therapy, day (mean ± *SD*)	9.5 (3–14)	0.5 (0–15)	**0.01**

COPD, chronic obstructive pulmonary disease; TCM, Traditional Chinese Medicine.

### Clinical symptoms

**Figure 1:**
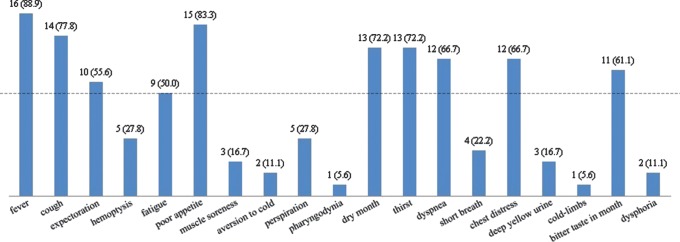
The distribution of clinical symptoms of 18 H7N9-infected patients.

As shown in Figure 1, more than half of the 18 H7N9-infected patients suffered from fever (88.9%), cough (77.8%), expectoration (55.6%), fatigue (50.0%), poor appetite (83.3%), dry month (72.2%), thirst (72.2%), dyspnea (66.7%), chest distress (66.7%), and bitter taste in month (61.1%). In addition, five patients suffered from hemoptysis and two suffered from dysphoria. Other symptoms such as muscle soreness (3), aversion to cold (2), perspiration (5), pharyngodynia (1), short breath (4), deep yellow urine (3), and cold-limbs (1) also occurred in patients with H7N9 infection.

### Laboratory features

Although no statistically significant differences were found in routine blood indices between the two groups, the C-reactive protein level was significantly higher in group B patients than in group A (Table 2). Also, serum creatinine level in group B was higher than that in group A (126.03±44.63 μmol/L in group B vs. 76.91±10.69 μmol/L in group A, *P* = 0.04), indicating more severe renal impairment in critically ill patients. While critically ill patients had significantly higher myoglobin levels than recovered patients (*P* = 0.016), no differences in immunoglobulin concentrations were observed between the two groups. However, all T cell subsets examined including total CD3^+^, CD4^+^, CD8^+^, and CD45^+^ T cells, were lower in group B patients than in group A.

**Table 2:** Laboratory tests of 18 H7N9-infected patients.

	Group A	Group B	
Laboratory tests	N = 10	N = 8	*P*
Blood routine			
White blood cell (10^9^/L)	4.835(1.99–8.74)	6.035(5.38–27.42)	0.374
Red blood cell (10^12^/L)	4.39±0.54	3.78±0.83	0.117
Hemoglobin (g/L)	137.7±13.46	114.5±23.53	0.097
Platelet cell (10^9^/L)	122.7±55.41	111.25±40.19	0.738
Neutrophils (10^9^/L)	3.66(1–8)	5.64(4–25)	0.477
Lymphocytes (10^9^/L)	0.63(0.46–1.2)	0.475(0.13–1.14)	0.894
Monocytes (10^9^/L)	0.18(0.07–0.59)	0.37(0.13–1.17)	0.374
Inflammation index			
C-reactive protein (mg/L)	54.99±38.6	109.38±20.28	**0.011**
Procalcitonin	0.12(0.04–7.72)	0.425(0.2–1.75)	0.143
Liver function			
Alanine aminotransferase (U/L)	57.5±28.14	31±5.89	0.097
Aspartate aminotransferase (U/L)	81.7±43.27	79.0±85.86	0.709
Lactate dehydrogenase (U/L)	504.1±197.52	718.25±456.71	0.157
Total bilirubin (μmol/L)	8.98±3.74	14.18±8.28	0.084
Direct bilirubin (μmol/L)	4.66±1.41	10.33±7.58	0.118
Albumin (g/L)	33.49±2.52	33.2±7.44	0.597
Globulin (g/L)	25.2±4.87	26.5±5.57	0.464
Renal function			
Blood ureanitrogen (mmol/L)	6.875±3.17	9.9±6.72	0.298
Serum creatinine (μmol/L)	76.91±10.69	126.03±44.63	**0.04**
Myocardial enzymes, (U/L)			
Creatine kinase	217.5(19–1854)	288(170–772)	0.424
Creatine kinase-MB isoenzyme	22.7±12.75	23.25±16.15	0.203
Myoglobin (ng/ml)	89.6±56.49	215.0±53.71	**0.016**
Pro-brain natriuretic peptide (pg/ml)	324.5(60–2582)	863(206–7286)	0.129
T cells, (cells/ul)			
CD3+	502.8±272.6	195±107.6	**0.033**
CD4+	345.8±194.6	143.8±94.3	**0.059**
CD8+	149±80.3	50.3±15.8	**0.015**
CD45+	797.0±356.0	416.0±194.8	**0.039**
Immunoglobulin, (g/L)			
IgA	2.19(1.6–5.02)	2.22(0.58–2.56)	0.884
IgG	13.73±4.78	13.18±3.4	0.698
IgM	1.15(0.61–2.02)	1.47(0.29–17.01)	0.696

### Factors associated with critical outcome

Baseline characteristics were included in the univariate analysis to identify possible factors associated with critical outcomes (Table 3). Our univariate analysis indicated that 1 mg/l increase in C-reactive protein (*P* = 0.032), 1 μmol/l increase in total bilirubin (*P* = 0.096), 1 μmol/l increase in serum creatinine (*P* = 0.1), 1 mg/l increase in myoglobin (*P* = 0.041), and 1 cell/μl increase in CD3^+^/CD4^+^/CD45^+^ T cells were likely associated with a critical outcome (*P* = 0.089, 0.093, and 0.08, respectively). One-day increase in TCM therapy was found to be significantly associated with a critical outcome (*P* = 0.017). These results might be limited by the small sample size in our study. Further studies with larger sample sizes should be conducted to verify our preliminary results.

**Table 3:** Univariable logistic regression model of critically ill condition of 18 H7N9-infected patients.

Covariates	OR	95%CI	*P*
C-reactive protein, per each increase of 1 mg/L	1.042	1.004–1.081	0.032
Total bilirubin, per each increase of 1 μmol/L	1.199	0.969–1.483	0.096
Serum creatinine, per each increase of 1 μmol/L	1.04	0.992–1.09	0.1
Myoglobin, per each increase of 1 mg/L	1.021	1.001–1.041	0.041
CD3+T cell, per each increase of 1 cell/ul	0.99	0.979–1.001	0.089
CD4+T cell, per each increase of 1 cell/ul	0.99	0.979–1.002	0.093
CD45+T cell, per each increase of 1 cell/ul	0.995	0.989–1.001	0.08
Duration of TCM therapy, per each increase of 1 day	0.707	0.532–0.94	0.017

OR, Odd Ratio; CI, Confidence Interval.

## Discussion and Conclusion

The recent discovery of H7N9 virus infections by a new influenza virus strain in three critically ill patients, as reported by Gao et al. [8], [10], is of major public health significance. Laboratory testing conducted in China showed that H7N9 viruses were sensitive to anti-influenza drugs known as neuraminidase inhibitors. These drugs were effective against seasonal influenza and H5N1 virus infections when given early in the course of the illness. However, these drugs had not yet been used to treat H7N9 infections.

To date, the National Health and Family Planning Commission of P. R. China has released two editions of the influenza A H7N9 clinic programs in which supporting therapies including oxygen therapy, antipyretic therapy, and cough and phlegm relief and medicinal therapy with antiviral drugs such as oseltamivir and zanamivir were recommended. Particularly, respiratory support including extracorporeal membrane oxygenation was recommended for critically ill patients. TCM therapy was also recommended as a second-line treatment for H7N9 infection. Previous studies have demonstrated that TCMs may have beneficial effects on patients with avian influenza [11], [12]. Our univariate logistic regression analysis indicated that the duration of TCM therapy could be partially associated with the prognosis in H7N9-infected patients. However, TCM therapy was not considered for four critically ill patients with ARDS, which might have accounted for the short duration of TCM therapy in the critically ill group. Consequently, the therapeutic value of TCM therapy in critically ill patients might have been underestimated. Further studies including randomized controlled trials are needed to fully evaluate TCM therapy in this patient population. Nine patients were also treated with intravenous immunoglobulins (IVIG). Six of the nine patients recovered, one was in severe condition, and two died.

Examination of baseline characteristics of H7N9-infected patients revealed that most patients were elderly males, suggesting that elderly people were at higher risk for H7N9 infection. In contrast, a high proportion of severe cases and deaths in H5N1 infection occurred in children [13]. The high proportion of elderly patients in H7N9 infection might be attributed to higher exposure of the elderly to H7N9 virus or to age-related body functions, such as decreased immune function [14]. Although H7N9 infections preferentially occurred in males, our logistic regression analysis did not show any statistically significant differences in clinical outcomes between genders.

Our results suggested that history of smoking and/or underlying clinical conditions may increase risk for infection, especially for patients with hypertension, diabetes, and heart–pulmonary insufficiency. Four patients, including three from group A and one from group B, had a smoking history of 30 to 40 years with 20 to 40 cigarette smoking per day, which could have contributed to their poor respiratory function. Nearly half of the patients (8/18) had hypertension, two had diabetes, three had coronary heart diseases, and three had COPD, indicating that chronic heart and lung diseases would increase risk for H7N9 infection.

Poultry and wild birds have been recognized as natural reservoir of IAVs [15]. In our study, only three patients had confirmed exposure to poultry or wild birds 7 d before onset of illness. However, according to previous report, 60% of H7N9-infected patients in China had epidemiological links to poultry and/or wild birds [16]. Li et al. also reported that 77% of H7N9-infected patients in their study had a history of exposure to live animals, including chicken (76%) [17]. Taken together, avoiding contact with poultry and wild birds may effectively reduce the risk of infection. Meanwhile, placing infected poultry and wild birds in quarantine is important for the control and prevention of H7N9 infection.

Our results also suggested that early diagnosis and treatment of H7N9 infection were critical in achieving favorable clinical outcomes. In our study, nearly all H7N9-infected patients were treated as common pneumonia in the early stage of the illness. The average time from onset of symptoms to H7N9 virus confirmation was 7.1±2.03 days in group A and 6.38±2.07 days in group B. Antiviral treatment was not initiated until 5–6 days after the onset of illness. The delayed diagnosis and treatment for this highly pathogenic avian influenza virus infection might have partially contributed to the poor prognosis in H7N9-infected patients. We believe that early surveillance and early diagnosis are of great importance for favorable clinical outcome. Full characterization of clinical features of H7N9 infection should speed up diagnosis and treatment.

Most H7N9-infected patients in our study had developed severe conditions with one or more complications, such as acute respiratory distress syndrome, heart failure, renal failure, septic shock, encephalopathy, and secondary infections. Our results suggested that such complications significantly worsened the clinical outcomes of H7N9-infected patients. Effective management of complications in the course of the disease would be critical to achieve favorable clinical outcomes and prevention of death.

Our results showed that H7N9-infected patients in critical conditions had higher C-reactive protein level and more severe renal insufficiency and myocardium injury. C-reactive protein level might be used as an early marker for clinical prognosis in H7N9 patients based on results from our logistic regression analyses, which was in agreement with previous studies showing that C-reactive protein level might serve as a marker for severity of illness in influenza infections [18], [19]. Since multivariate analysis was not performed in our small sample study, further research is needed to verify this.

Interestingly, levels of T cell subsets including total CD3^+^, CD4^+^, CD8^+^, and CD45^+^ T cells, were all lower in patients in critical conditions than those in recovered conditions. The protective role of CD8^+^ T cells in AIV infections has been discussed in previous studies [20], [21]. It has been shown that CD8^+^ T cell-mediated responses in patients were mainly directed against conserved viral proteins, enabling CD8^+^ T cells to provide protection against heterologous influenza strains [22]. CD4^+^ T cells have also been reported to contribute to immunity against influenza. Previous studies showed that CD4^+^ T cells were induced after influenza virus infection and played a central role in acquired immunity through induction and maintenance of CD8^+^ memory T cells and assisting B cells in antibody production [23], [24]. CD45^+^ T cells were found to be not only essential for efficient T- and B-cell antigen receptor signal transduction but also involved in cytokine signaling [25], [26]. CD45KO mice and humans who lacked CD45 expression were severely immunodeficient and had very few peripheral T lymphocytes with impaired T and B cell responses [27]–[29]. Results of our analysis suggested that T cell-mediated responses might play an important role in immune defense against H7N9 virus infection. Further studies on T cell-mediated responses during H7N9 infection might provide useful information to help develop effective therapies such as H7N9 vaccines.

We used glucocorticoid therapy for most H7N9-infected patients in our observational study. Some controversies exist in the use of glucocorticoid therapy for IAV infection. Han et al. reported that early use of parenteral glucocorticoid therapy for fever reduction and pneumonia prevention in patients with pandemic influenza A (H1N1) infection increased the risk of critical disease and death [30]. Likewise, He et al. advised that glucocorticoids should not be used in critically ill patients with H1N1 influenza infection [31]. However, Carter argued that while steroids should not be used as monotherapy in the treatment of avian influenza, they might provide therapeutic benefits as an adjunct therapy to antiviral agents if prescribed at appropriate dosages [32]. We found no clear association between glucocorticoid therapy and clinical outcomes in H7N9-infected patients in our study. Future studies with larger patient populations are required to evaluate the benefits and risks asscocaited with glucocorticoid therapy in treatment of H7N9 infections.

In conclusion, successful treatment of H7N9 influenza depends on early diagnosis of H7N9 infection at the onset of clinical symptoms even if they are mild. Knowledge of risk factors, clinical features, and potential complications discussed in our paper would help clinicians who triage patients with suspected or confirmed H7N9 infection determine appropriate treatment strategies and fight potential epidemic/pandemic. Increased research and surveillance are required to further understand the pathogenesis of H7N9 infection and epidemiological factors that contribute to severe conditions. Further studies are also needed to identify H7N9 natural reservoirs and delineate mechanisms of transmission.

The preliminary results reported in the present article were from a small sample study. Further studies with larger sample sizes are needed to verify and extend our findings.
